# Inactivation of ribosomal protein S27-like impairs DNA interstrand cross-link repair by destabilization of FANCD2 and FANCI

**DOI:** 10.1038/s41419-020-03082-9

**Published:** 2020-10-13

**Authors:** Siyuan Sun, Hengqian He, Yuanyuan Ma, Jie Xu, Guoan Chen, Yi Sun, Xiufang Xiong

**Affiliations:** 1 Cancer Institute of the Second Affiliated Hospital, and Institute of Translational Medicine, Zhejiang University School of Medicine, Hangzhou, Zhejiang P. R. China; 2 Shanghai Institute of Precision Medicine, Ninth People’s Hospital, Shanghai Jiao Tong University School of Medicine, Shanghai, China; 3 Department of Urology, the Second Affiliated Hospital, Third Military Medical University (Army Medical University), Chongqing, P. R. China; 4 School of Medicine, Southern University of Science and Technology, Shenzhen, Guangdong P. R. China

**Keywords:** Ribosomal proteins, Lysosomes

## Abstract

Ribosomal protein S27-like (RPS27L), an evolutionarily conserved ribosomal protein and a direct p53 target, plays an important role in maintenance of genome integrity. We have previously reported that RPS27L regulates radiation sensitivity via the MDM2-p53 and MDM2-MRN-ATM axes. Whether and how RPS27L modulates DNA interstrand cross-link (ICL) repair is unknown. Here we identified that RPS27L binds to FANCD2 and FANCI, two Fanconi anemia (FA) proteins functioning in ICL repair pathway. Upon RPS27L knockdown, the levels of FANCD2 and FANCI are reduced due to accelerated degradation via p62-mediated autophagy-lysosome pathway, which is abrogated by chloroquine (CQ) treatment or Beclin 1 knockdown. Biologically, RPS27L knockdown suppresses FANCD2 foci formation and impairs ICL repair upon exposure to ICL-inducing agent mitomycin C (MMC) in lung cancer cells. This effect of MMC sensitization can be partially reversed by CQ treatment. Together, our study shows that RPS27L positively regulates ICL repair by binding with FANCD2 and FANCI to prevent their degradation via autophagy-lysosome system.

## Introduction

DNA interstrand cross-links (ICLs), an extremely deleterious form of DNA lesions, are induced by widely used chemotherapeutic agents, such as mitomycin C (MMC), cisplatin, and psoralen, as well as by endogenous metabolic intermediates, such as aldehydes^1,2^. ICLs covalently link the two strands of DNA to impede DNA replication and transcription by blocking strand separation^3,4^. Mechanistically, ICLs are detected and repaired by Fanconi anemia (FA) pathway, which coordinates other DNA repair pathways, including homologous recombination, nucleotide excision repair, and translesion synthesis, to maintain genomic stability^1^. Thus, deficiency in the FA pathway by germline mutations in any of FA genes, occurred in patients with Fanconi anemia^5^, causes genomic instability and high predisposition to various cancers.

Of 22 FA proteins, FANCD2 and FANCI, which form a heterodimer with each other, known as ID2 complex, appear to play a central role in the FA pathway to repair ICLs. Specifically, ID2 complex is recruited to chromatin at DNA ICLs, where both FANCD2 and FANCI are monoubiquitylated by the FA core complex, consisting of multiple FA proteins (FANCA/B/C/E/F/G/L/M) and other FA-associated proteins^1^. Next, the monoubiquitylated ID2 complex recruits downstream repair effectors, such as structure-specific nucleases, to unhook and excise the ICLs, and to eventually repair the ICLs^6,7^. Upon completion of DNA repair, the ID2 complex is deubiquitinated by the USP1-UAF1 deubiquitinase complex^8^.

Ribosomal protein S27-like (RPS27L, NM_015920), an evolutionarily conserved ribosomal protein^9^, plays an important role in maintenance of genome integrity. RPS27L was first identified as a direct p53 transcriptional target by He and Sun^10^ and others^11^. As an MDM2 binding protein, RPS27L knockdown reduced p53 levels by promoting MDM2-mediated p53 ubiquitylation in some cancer cell lines^9^. Our mouse *Rps27l* knockout study revealed several important physiological roles of Rps27l. First, *Rps27l* depletion induced p53 levels in primary mouse embryonic fibroblasts (MEFs) and multiple organs^12^. Second, *Rps27l* knockout accumulated p53 and promoted apoptosis of hematopoietic stem and progenitor cells, leading to growth retardation and postnatal death^12^. Third, *Rps27l* deletion in primary MEFs triggered genomic instability, when one allele of p53 was simultaneously deleted^12^, which conferred selection pressure for the loss of p53 heterozygosity, leading to lymphomagenesis in vivo^12^. Fourth, the *Rps27l*^*−/−*^*;Trp53*^*+/−*^ mice were extremely sensitive to radiation by modulating the Mdm2-p53 and Mdm2-MRN-ATM axes^13^. Specifically, *Rps27l* disruption impaired DNA damage response by increasing Mdm2 binding of Nbs1 to inhibit Nbs1-mediated Atm activation^13^. However, whether and how RPS27L regulates DNA ICL repair is previously unknown.

Here we report that RPS27L physically bound to FANCD2 and FANCI, and RPS27L knockdown reduced the protein levels of FANCD2 and FANCI by promoting their degradation via p62-mediated autophagy-lysosome pathway. RPS27L knockdown, therefore, impaired ICL repair and enhanced the sensitivity of lung cancer cells to ICL-inducing agent MMC. Thus, our study revealed a novel function of RPS27L in regulation of ICL repair by stabilization of FANCD2 and FANCI, and suggested that RPS27L might serve as an attractive target for the sensitization of ICL-inducing chemotherapy.

## Results

### RPS27L interacts with FANCD2 and FANCI

**Fig. 1:**
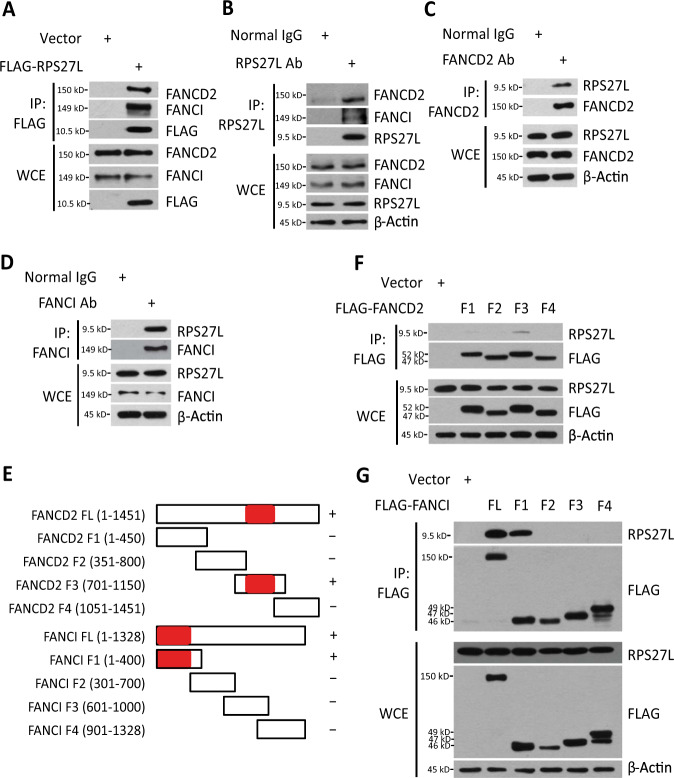
RPS27L interacts with FANCD2 and FANCI. **A** Ectopically expressed RPS27L binds to endogenous FANCD2 and FANCI. H1299 cells were transfected with mock vector or FLAG-RPS27L, and subjected to immunoprecipitation (IP) with FLAG beads, followed by immunoblotting (IB) with indicated antibodies (Abs). WCE: whole-cell extract. **B**–**D** Endogenous RPS27L binds to endogenous FANCD2 and FANCI. A549 cells were harvested for IP with RPS27L (**B**), FANCD2 (**C**), or FANCI (**D**) Ab, and then IB with indicated Abs. **E** Schematic diagrams of full length and fragments of FANCD2 and FANCI constructs. The bar graphs were not drawn to scale and the red bar indicates the RPS27L-interacting region. **F**, **G** Mapping of FANCD2/FANCI binding to RPS27L. 293 cells were transfected with mock vector, indicated constructs encoding various fragments of FLAG-FANCD2 (**F**) or FLAG-FANCI (**G**) before harvesting for IP with FLAG beads, followed by IB with indicated Abs.

Our previous study suggested that RPS27L regulates radiation sensitivity in both p53-dependent and p53-independent manners^13^. To further elucidate the mechanism by which RPS27L regulates genomic stability, we first identified RPS27L putative binding proteins by mass spectrometry and focused on FANCD2 and FANCI (hereafter FANCD2/FANCI), two proteins known to be involved in DNA damage and repair. We then performed co-immunoprecipitation experiments to verify the interaction between FANCD2/FANCI and RPS27L and found that ectopically expressed FLAG-RPS27L readily pulled down endogenous FANCD2 and FANCI (Fig. 1A). More significantly, endogenous RPS27L also formed a complex with FANCD2/FANCI via the two-way immunoprecipitation (Fig. 1B–D). To further define the binding regions on FANCD2 and FANCI responsible for their interaction with RPS27L, we generated a series of FLAG-tagged FANCD2 and FANCI fragments (Fig. 1E), and determined their binding ability to endogenous RPS27L. We found that FANCD2 fragment (701–1105), but not other fragments, including the N-terminal portion (1–450), fragment (351–800) or the C-terminal portion (1051–1451), bound to endogenous RPS27L (Fig. 1F), indicating that the binding region on FANCD2 is from residues 801 to 1050. Likewise, full-length FANCI and N-terminal region (1–400), but not other fragments, including fragment (301-700), fragment (601-1000), or the C-terminal portion (901–1328), pulled down endogenous RPS27L (Fig. 1G), indicating that RPS27L binds to N-terminal portion of FANCI (1–300). Taken together, both FANCD2 and FANCI bind to RPS27L, and their binding regions to RPS27L are from residues 801 to 1050 on FANCD2, and from residues 1 to 300 on FANCI, respectively.

### RPS27L knockdown reduces the protein levels of FANCD2 and FANCI

**Fig. 2:**
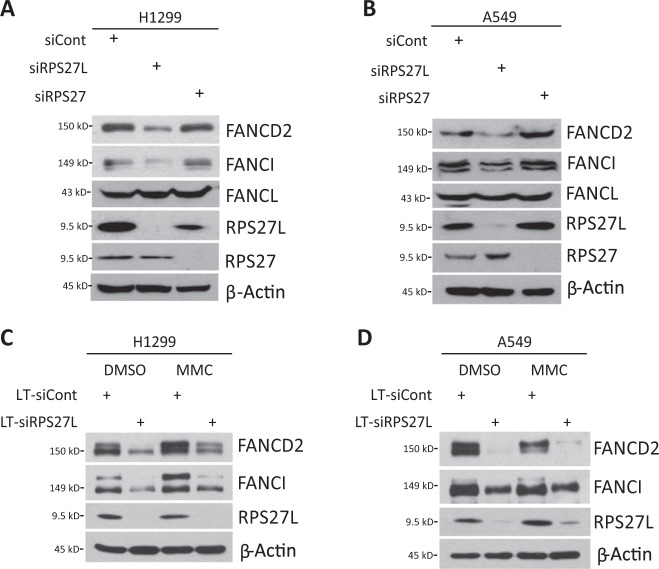
RPS27L knockdown specifically decreases the levels of FANCD2 and FANCI. **A**, **B** RPS27L knockdown reduces the protein levels of FANCD2 and FANCI. H1299 (**A**) and A549 (**B**) cells were transfected with siRNA oligos targeting RPS27L (siRPS27L) or RPS27 (siRPS27), or scrambled control siRNA (siCont). After 48 h, cells were harvested for IB with indicated Abs. **C**, **D** RPS27L knockdown reduces the protein levels of FANCD2 and FANCI upon MMC treatment. H1299 (**C**) and A549 (**D**) cells were infected with indicated lentivirus for 72 h, and then left untreated or treated with 1 μM MMC for additional 24 h, followed by IB with indicated Abs. The upper band of FANCD2/FANCI is the monoubiquitylated form, and the lower band is the nonubiquitylated form.

We next determined the biochemical consequence of RPS27L binding of FANCD2/FANCI via knockdown approach. The lentivirus-based siRNA silencing of RPS27L, but not its family protein RPS27, which differs from RPS27L only by three amino acids at its N-terminus^9^, specifically decreased the protein levels of endogenous FANCD2 and FANCI in both H1299 and A549 cells (Fig. 2A, B). Serving as a negative control, the levels of FANCL, one component of the FA core complex, had minimal, if any, change upon silencing of either RPS27L or RPS27 (Fig. 2A, B). Consistent with the reduced abundance of FANCD2 and FANCI, their monoubiquitylation in response to the treatment of MMC, an ICL-inducing agent, was also reduced in H1299 and A549 cells (Fig. 2C, D), suggesting that RPS27L regulates the levels of total FANCD2 and FANCI proteins. Collectively, these results suggest that RPS27L silencing downregulates the levels of FANCD2 and FANCI selectively.

### RPS27L knockdown destabilizes FANCD2 and FANCI via p62-mediated autophagy-lysosome pathway

**Fig. 3:**
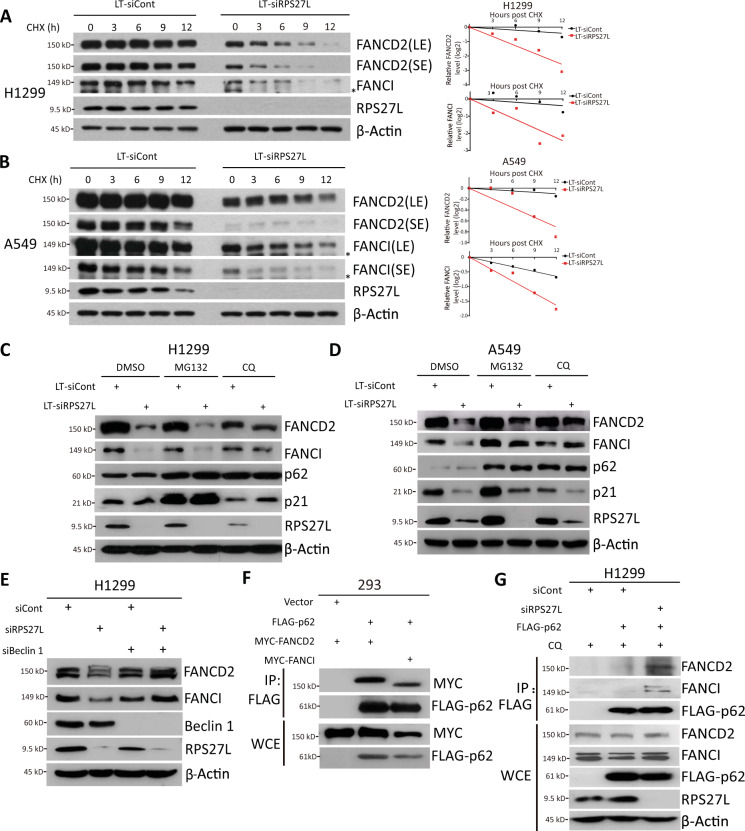
RPS27L knockdown destabilizes FANCD2 and FANCI via p62-mediated autophagy-lysosome pathway. **A**, **B** Silencing of RPS27L shortens the protein half-lives of FANCD2 and FANCI protein. H1299 (**A**) and A549 (**B**) cells were infected with indicated lentivirus for 48 h, followed by CHX treatment for indicated periods of time before harvesting for IB with indicated Abs (left). Densitometry quantification was performed with ImageJ, and the decay curves are shown (right). LE: longer exposure; SE: shorter exposure. * Non-specific band. **C**, **D** Chloroquine (CQ), but not MG132, abrogates FANCD2 and FANCI reduction induced by RPS27L knockdown. H1299 cells (**C**) and A549 cells (**D**) were infected with indicated lentivirus, and then harvested for IB after treatment with MG132 (1 µM) for 12 h or CQ (50 µM) for 24 h. **E** Beclin 1 knockdown abrogates FANCD2/FANCI reduction upon RPS27L knockdown. H1299 cells were transfected with indicated siRNA oligos, and then harvested for IB. **F** FANCD2/FANCI binds to p62. 293 cells were transfected with indicated plasmids for 48 h, and then IP with FLAG beads before harvesting for IB with indicated Abs. WCE: whole-cell extract. **G** RPS27L knockdown promotes FANCD2/FANCI binding to p62. H1299 cells were transfected with indicated siRNA oligos. After 24 h, cells were transfected with mock vector or FLAG-p62 for additional 48 h, and then treated with CQ (50 µM) for 24 h before harvesting for IP with FLAG beads, followed by IB with indicated Abs. The upper band of FANCD2/FANCI is the monoubiquitylated form, and the lower band is the nonubiquitylated form.

Having established that RPS27L knockdown reduced the levels of FANCD2/FANCI, we next determined whether RPS27L regulates the levels of both proteins at the post-translational levels. We measured the half-lives of both proteins in the presence of cyclohexamide (CHX), an inhibitor to block new protein synthesis, and found that RPS27L knockdown significantly shortened the protein half-lives of FANCD2 and FANCI in both H1299 (Fig. 3A) and A549 cells (Fig. 3B). We further determined whether the reduction of FANCD2 and FANCI upon RPS27L knockdown would be rescued by MG132, a proteasome inhibitor, or chloroquine (CQ), a lysosomal inhibitor that impairs autophagosome fusion with lysosomes^14^. Notably, the reduction of FANCD2 and FANCI was largely rescued by CQ, whereas MG132 had minor, if any, rescuing effect (Fig. 3C, D). The levels of p21 and p62, served as positive controls, were accumulated upon the treatment of MG132 or CQ in cells transfected with scramble-control siRNA oligos, respectively (Fig. 3C, D). Moreover, knockdown of Beclin 1, a central regulator of autophagy for autophagosome formation and maturation^15^, also abrogated the FANCD2/FANCI reduction induced by RPS27L knockdown (Fig. 3E). Taken together, the rescuing effect by both pharmacological and genetic approaches indicates that the autophagy-lysosome system plays a causal role in the destabilization of FANCD2 and FANCI triggered by RPS27L knockdown.

To further define the mechanism of FANCD2/FANCI reduction via the autophagy-lysosome system, we determined if FANCD2/FANCI would bind to p62/SQSTM1, a key receptor to mediate protein clearance via autolysosome^16^. We first co-transfected MYC-tagged FANCD2 and FANCI with FLAG-p62, and found that ectopically expressed p62 pulled down both ectopically expressed FANCD2 and FANCI (Fig. 3F), suggesting that p62 might mediate the degradation of both FA proteins via autolysosome. More importantly, RPS27L knockdown promoted the binding of p62 with endogenous FANCD2 and FANCI (Fig. 3G). Taken together, RPS27L knockdown destabilizes FANCD2 and FANCI by shortening their protein half-lives via p62-mediated autophagy-lysosome pathway.

### RPS27L knockdown impairs ICL repair and sensitizes cells to MMC

**Fig. 4:**
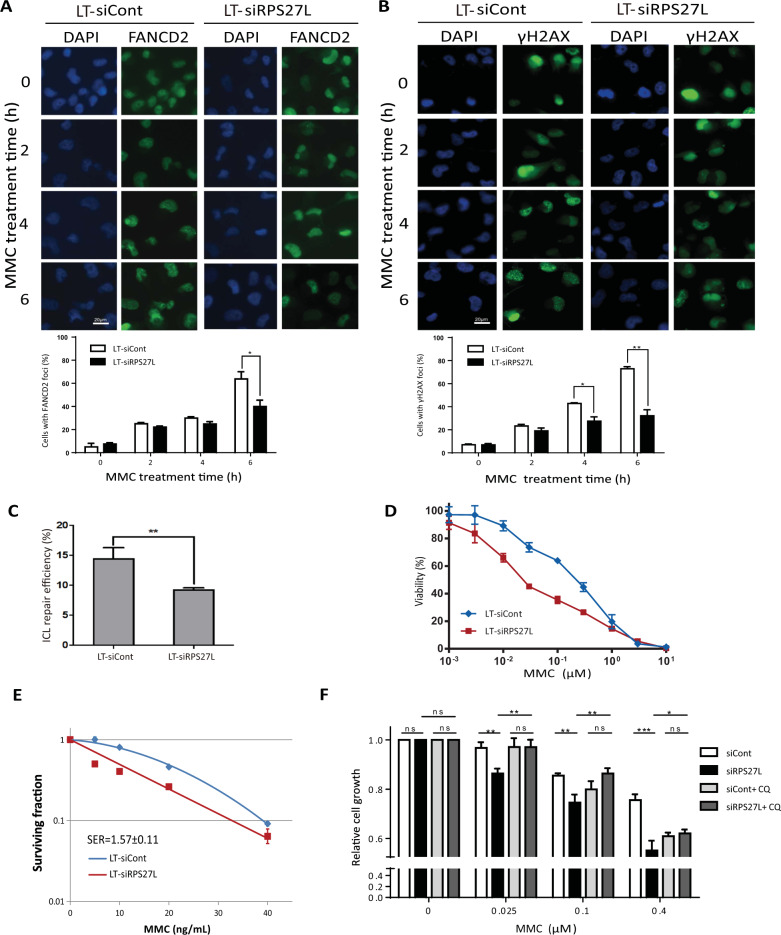
RPS27L knockdown impairs ICL repair and triggers MMC sensitization. **A**, **B** RPS27L knockdown reduces ICL repair upon MMC treatment. A549 cells, infected with indicated lentivirus, were treated with MMC at 1 µM for indicated periods of time, followed by immunofluorescent staining with FANCD2 (**A**) or γH2AX (**B**) Ab (top). Scale bar = 20 μm. The cells with foci in five random fields were counted (bottom). Shown are mean ± SEM from three independent experiments; *p < 0.05; **p < 0.01. **C** RPS27L knockdown impairs ICL repair. H1299 cells, infected with indicated lentivirus, were transfected with plasmid reporter substrates with a site-specific ICL for 24 h, and then subjected to luciferase reporter reactivation assay. Shown are mean ± SEM from three independent experiments; **p < 0.01. **D**, **E** RPS27L knockdown sensitizes lung cancer cells to MMC treatment. A549 cells were infected with indicated lentivirus for 48 h, and then treated with various concentrations of MMC for 48 h, followed by MTT assay (**D**). H1299 cells were infected with indicated lentivirus, and then treated with MMC for 24 h, followed by clonogenic survival assay (**E**). Shown are mean ± SEM from three independent experiments. **F** CQ treatment partially abrogates MMC sensitization upon RPS27L knockdown. A549 cells were transfected indicated siRNA oligos for 48 h, and then treated with various concentrations of MMC in the absence or presence of CQ (25 µM) for 24 h, followed by CCK-8 assay. Shown are mean ± SEM from three independent experiments; *p < 0.05, **p < 0.01, ***p < 0.001, ns, no significance.

Given the crucial role of FANCD2 and FANCI in the FA pathway to repair DNA ICLs, we next examined if RPS27L knockdown would affect their foci formation at DNA damaged site in response to ICL-inducing agent MMC. Indeed, upon MMC treatment, the formation of FANCD2 foci was significantly reduced by RPS27L knockdown (Fig. 4A). Consistently, phosphorylation of H2AX at Ser 139 (γH2AX), a sensitive marker to detect DNA double-strand breaks which are also generated in the process of ICL repair^1^, was also decreased with RPS27L knockdown (Fig. 4B), indicating a reduced ICL repair upon RPS27L knockdown. Moreover, using an ICL repair assay based on dual-luciferase reporter system^17^, we found that the ICL repair efficiency was significantly lower upon RPS27L knockdown (Fig. 4C). As a result, cell viability (Fig. 4D) and colony formation (Fig. 4E) after MMC treatment were significantly decreased by RPS27L knockdown, suggesting an increased sensitivity to MMC in RPS27L knockdown cells. Given that CQ partially blocks the reduction of FANCD2 and FANCI by RPS27L knockdown, we determined if CQ would rescue MMC sensitization. Indeed, MMC-induced inhibition of cell growth under RPS27L knockdown condition was partially rescued by CQ treatment (Fig. 4F). Taken together, our study strongly suggests that RPS27L plays an important role in ICL repair by protecting FANCD2 and FANCI from degradation via the autophagy-lysosome system, and is responsible for cellular sensitivity to MMC.

## Discussion

Our recent study showed that RPS27L regulates DNA damage response by modulating the MDM2-MRN-ATM axis^13^. An earlier study reported a role of RPS27L in DNA repair, based upon the observation that RAD51 and PRKDC, two DNA DSB repair genes were transcriptionally decreased upon RPS27L knockdown in response to DNA damage^18^. Here we reported that RPS27L also regulates ICL repair via stabilization of FANCD2 and FANCI in lung cancer cells. Our conclusion is based upon the following lines of evidence: (1) Ectopically expressed or endogenous RPS27L interacts with endogenous FANCD2 and FANCI (Fig. 1); (2) RPS27L knockdown via lenti-viral or siRNA approach specifically reduces the levels of FANCD2 and FANCI in the absence and presence of MMC (Fig. 2); (3) RPS27L knockdown impairs the formation of FANCD2 foci and decreases ICL repair efficiency upon MMC treatment (Fig. 4A, C); (4) RPS27L knockdown inhibits cell viability and colony formation after MMC treatment in lung cancer cells (Fig. 4D, E). In fact, several ribosomal proteins, including RPS3^19,20^, RPL3^21^, RPL6^22^, and RPL26^23^, have also been reported to directly participate in DNA damage response and repair to ensure genomic integrity through multiple mechanisms^24,25^. For example, RPL6 directly bound to histone H2A in response to DNA damage, and RPL6 silencing impairs DNA damage-induced H2A/H2AX ubiquitination and the interaction between MDC1 and γH2AX, resulting in defects in DNA repair and reduced cell survival^22^. Thus, our study adds RPS27L into the list of ribosomal proteins in direct regulation of DNA damage response and DNA repair, uniquely in regulation of the ICL repair.

Although RPS27L binds to FANCD2 and FANCI, RPS27L appears not to affect their monoubiquitylation. Reduced levels of monoubiquiylated FANCD2/FANCI upon RPS27L knockdown after exposure to MMC (Fig. 2) is likely due to overall reduction of FANCD2/FANCI protein levels, since the binding regions of RPS27L on FANCD2 (residues 801–1050) and FANCI (residues 1–300) (Fig. 1E–G) exclude the monoubiquitylation sites at Lys 561 on FANCD2^26^ and Lys 523 on FANCI^27^, respectively. It is worth noting that while the alignment of amino acid sequence on FANCI and FANCD2 for RPS27L binding shared the low similarity^28^, the binding region of RPS27L on FANCD2 is a portion of the ARM repeats, which is known to mediate the protein-protein interaction^29^. Lack of a complex structure of the RPS27L-FANCI or RPS27L-FANCD2 prevented us to provide a solid explanation as to how RPS27L binds to these two regions, respectively. Furthermore, it appears that RPS27L-FANCD2/FANCI binding and FANCD2/FANCI stabilization are rather specific, since RPS27, a RPS27L family member, failed to affect the levels of FANCD2/FANCI, whereas RPS27L failed to reduce the level of FANCL, the ubiquitin E3 ligase component in the FA core complex that catalyzes the monoubiquitylation of FANCD2 and FANCI^30^ (Fig. 2).

We also made a novel observation that knockdown of RPS27L, but not its family member RPS27, destabilizes FANCD2 and FANCI by accelerating their degradation via lysosomal system (Fig. 3). It has been previously reported that the stability of FANCD2 is regulated via both proteasome and caspase-3 pathways^31,32^. MG132 restores the reduction of FANCD2 levels upon FANCJ depletion^32^. However, we found that the FANCD2 and FANCI reduction by RPS27L knockdown was not reversed by MG132, rather by either CQ, a lysosomal inhibitor, or knockdown of Beclin 1, a key regulator of autophagy (Fig. 3C–E). Thus, FANCD2 and FANCI degradation upon RPS27L knockdown is likely mediated by the autophagy-lysosome system, one of the two major protein degradation systems responsible for cellular homeostasis^33^. Moreover, p62, a selective autophagy receptor that targets cargoes to the autophagosomal membrane for autophagic degradation^34^, indeed bound with both FANCD2 and FANCI (Fig. 3F), and such binding was further promoted when RPS27L was knocked down (Fig. 3G), suggesting that RPS27L knockdown triggers degradation of FANCD2 and FANCI via p62-mediated autophagy-lysosome pathway. Consistently, we recently reported that RPS27L knockdown induces autophagy in breast cancer cells^35^, which could contribute to FANCD2/FANCI degradation.

It is known that RPS27L differs from its family member RPS27 (NM_001030) by only three amino acids (R5K, L12P, K17R) at the N-terminus^9^. The interaction network of RPS27 in the structures of ribosome (PDB code 5A2Q) show that its N-terminus is critical for protein-protein interaction. Indeed, our previous study showed that both RPS27L and RPS27 bind to MDM2, an E3 ubiquitin ligase, through their N-terminal 36 residues. However, the interaction affinity between RPS27L and MDM2 is stronger than that between RPS27 and MDM2^9^. Although the N-terminal sequence of RPS27L shares some similarity to the regions of p62 overlapping ZZ domain and UBA domain, which are involved in recognition of N-terminally arginylated substrates^36,37^ and binding to ubiquitylated cargoes^38–40^, respectively, whether and how RPS27L (via its N-terminal portion) modulates the p62-FANCs interaction via direct competition or by the other means need further investigation. Nevertheless, the fact that only RPS27L affects p62-mediated degradation of the FANCD2/FANCI, strongly suggested that the difference in these three amino acids at the N-terminus plays the determinant role.

Biologically, FANCD2 destabilization upon RPS27L knockdown caused decreased formation of FANCD2 foci as well as reduced formation of γH2AX foci (Fig. 4A, B), leading to sensitization of lung cancer cells to MMC. The partial blockage of MMC sensitization by CQ treatment (Fig. 4F) suggested a causal role of the RPS27L-FANCD2/FANCI axis in regulation of MMC sensitivity. Thus, our results suggest that the binding of RPS27L to FANCD2/FANCI may stabilize FANCD2 and FANCI by preventing their degradation via autophagy-lysosome system, which facilitates the formation of FANCD2 foci, ICL repair and cell survival upon MMC treatment. It is worth noting that the regulation of the proteasomal degradation of other FA proteins, such as FANCA^41^, FANCJ^42^, has also been reported to play an essential role in the functioning of FA pathway.

Two previous studies by our own laboratory and from another group showed that RPS27L expression was downregulated in human breast cancer^35^ and colorectal cancer^18^, respectively. Moreover, reduced RPS27L expression in either feces or colorectal cancer tissues was also significantly associated with poor survival of patients with colorectal cancer^18^. We found that RPS27L mRNA was also remarkably decreased in non-small cell lung cancer (NSCLC) tissues (Fig. S1A), which correlated with poor differentiation (Fig. S1B), indicating a potential tumor-suppressive role of RPS27L in NSCLC. Notably, the study with *Rps27l* knockout mice also highly suggests a suppressive role of Rps27l in spontaneous lymphomagenesis^12^. Given that RPS27L knockdown increases the sensitivity of lung cancer cells to MMC, and RPS27L expression is reduced in NSCLC tissues, our study suggests that ICL-inducing chemotherapeutic agents may have better therapeutic efficacy in the treatment of NSCLC patients with low RPS27L expression.

In summary, we reported here a novel connection between a ribosomal protein and Fanconi anemia proteins, demonstrating that RPS27L binds to and stabilizes FANCD2 and FANCI. Given that RPS27L knockdown destabilizes FANCD2 and FANCI via p62-mediated autophagy-lysosome pathway, leading to impaired ICL repair and sensitization of lung cancer cells to MMC, our study, therefore, suggests that RPS27L might serve as an attractive target for the sensitization of ICL-inducing chemotherapy.

## Materials and methods

### Cell culture and chemicals

H1299, A549, and HEK293 cells were obtained from American Type Culture Collection and cultured in Dulbecco’s modified Eagle’s medium supplemented with 10% (v/v) fetal bovine serum and 1% penicillin-streptomycin at 37 °C in a humidified incubator with 5% CO_2_. The following chemicals were obtained from commercial sources: mitomycin C (Cayman), MG132 (MedChemExpress), cyclohexamide and chloroquine (Sigma-Aldrich).

### Western blotting analysis and immunoprecipitation

Cells were harvested following various treatments, lysed in lysis buffer with phosphatase inhibitors (Roche), and subjected to western blotting analysis and immunoprecipitation as described in ref. ^43^, using various antibodies as follows: FANCD2 (Santa Cruz, sc-20022), FANCI (Bethyl, A300-212A), FANCI (Proteintech, 20789-1-AP), FANCL polyclonal rabbit antibody (gift from Dr. Weidong Wang), FLAG (Sigma-Aldrich, F1804), β-actin (Sigma-Aldrich, A5441), p62 (MBL Life science, PM045), p21 (Cell Signaling Technology, 2947S), and Beclin 1 (Cell Signaling Technology, 4122S). RPS27L and RPS27 polyclonal rabbit antibodies were raised and purified as described in ref. ^10^. Anti-FLAG M2 affinity gel (Sigma-Aldrich, A2220) were used to immunoprecipitate ectopically expressed FLAG-tagged proteins.

### Lentivirus and siRNA-based gene knockdown

The sequences of RPS27L/RPS27 siRNA oligonucleotides used for construction of lentivirus-silencing vector and for transfection were described previously^9,35^. The sequence of siRNA oligo targeting Beclin 1 is as follows: siBeclin 1: 5′-CGA CTT GTT CCT TAC GGA A-3′. Cells were infected with lentivirus or transfected with siRNA oligos using Genmute according to the manufacturer’s instructions (SignaGen Laboratories). After 48–72 h, the cells were split for assays.

### Immunofluorescence

Cells after infection with lentivirus-based shRNA for 48 h were treated with 1 μM MMC for various periods of time. Cells were then fixed and immunostained with anti-FANCD2 (Santa Cruz, sc-20022) or anti-γH2AX (Millipore, JBW301) antibody, followed by incubation with Alexa Fluor 488 conjugated secondary antibodies (Life Technologies). DAPI (Life Technologies) was used for nuclear counterstaining. Stained cells were examined under a fluorescence microscope (Olympus).

### Interstrand cross-link repair assay

The ICL repair assay was performed as previously described^17^. Briefly, the plasmid reporter substrates with a site-specific ICL (kindly provided by Dr. Lei Li), which is located between the CMV promoter and the initiation ATG of a firefly luciferase reporter, were transfected into cells. A renilla luciferase reporter plasmid was included in each sample as an internal control. Cells were subjected to luciferase reporter reactivation assay 24 h post transfection using dual-luciferase reporter assay system (Promega), according to the manufacturer’s instructions.

### Cytotoxicity and clonogenic survival assay

Cytotoxicity was evaluated by MTT assay or CCK-8 assay. For MTT assays, cells seeded in 96-well plates were treated with various concentrations of MMC, followed by incubation with MTT (Sigma) for 4 h at 37 °C. After incubation, DMSO was added into each well, and the absorbance at 570 nm was read. For CCK-8 assays, cell viability was evaluated using Cell Counting Kit-8 (MedChemExpress), according to the manufacturer’s instructions. For clonogenic survival assays, cells infected with indicated lentivirus were treated with MMC for 24 h. Cells were then split into 6-well plates and allowed to grow for 10 days. Colonies were fixed with methanol and stained with crystal violet, and colonies with more than 50 cells were counted. The results from three independent experiments, each run in triplicates were plotted^44^.

### Statistical analysis

The two-tailed student *t*-test for statistical analyses were performed using PRISM 5 (GraphPad). Data were expressed as mean ± SEM. Statistical significance was determined as *p* < 0.05.

## Supplementary information

Supplemental Materials
