# Supplementary material for: Inactivation of ribosomal protein S27-like impairs DNA interstrand cross-link repair by destabilization of FANCD2 and FANCI
Source: Cell Death Dis. 2020 Oct 13;11(10):852. doi: 10.1038/s41419-020-03082-9 (PMC7555897; doi:10.1038/s41419-020-03082-9)

Supplemental Materials

Figure Legends

Figure S1. RPS27L level is reduced in patients with lung cancer.

(A) RPS27L mRNA was lower in different types of lung cancer as compared to normal lung tissues in Hou's dataset. The relative RPS27L mRNA value was normalized using Robust Multi-Array Average (RMA) method and log 2 transformed data. t-test, $p <0.01$. N: normal lung tissues, ADC: lung adenocarcinoma, LCC: large cell lung cancer, SCC: squamous cell lung cancer. (B) RPS27L mRNA was lower in poorly differentiated lung tumors as compared to well differentiated tumors in Shedden's dataset. t-test, $p <0.01$.

Materials and Methods

Primary tumor-derived gene expression microarray datasets

The published Affymetrix microarray datasets (1, 2) were downloaded. The CEL files of microarray data were normalized using Robust Multi-Array Average (RMA) method (3) and log 2 transformed data were used. Boxplot was used to show the different RPS27L mRNA expression in lung cancers and normal tissues, as well as in tumor differentiation.

References

1. Director's Challenge Consortium for the Molecular Classification of Lung, A., Shedden, K., Taylor, J. M., Enkemann, S. A., Tsao, M. S., Yeatman, T. J., Gerald, W. L., Eschrich, S., Jurisica, I., Giordano, T. J., Misek, D. E., Chang, A. C., Zhu, C. Q., Strumpf, D., Hanash, S., Shepherd, F. A., Ding, K., Seymour, L., Naoki, K., Pennell, N., Weir, B., Verhaak, R., Ladd-Acosta, C., Golub, T., Gruidl, M., Sharma, A., Szoke, J., Zakowski, M., Rusch, V., Kris, M., Viale, A., Motoi, N., Travis, W., Conley, B., Seshan, V. E., Meyerson, M., Kuick, R., Dobbin, K. K., Lively, T., Jacobson, J. W., and Beer, D. G. (2008) Gene expression-based survival prediction in lung adenocarcinoma: a multi-site, blinded validation study. *Nat Med* **14**, 822-827
2. Hou, J., Aerts, J., den Hamer, B., van Ijcken, W., den Bakker, M., Riegman, P.,

van der Leest, C., van der Spek, P., Foekens, J. A., Hoogsteden, H. C., Grosveld, F., and Philipsen, S. (2010) Gene expression-based classification of non-small cell lung carcinomas and survival prediction. *PLoS One* **5**, e10312

3. Irizarry, R. A., Hobbs, B., Collin, F., Beazer-Barclay, Y. D., Antonellis, K. J., Scherf, U., and Speed, T. P. (2003) Exploration, normalization, and summaries of high density oligonucleotide array probe level data. *Biostatistics* **4**, 249-264

Sun et al. Figure S1

A

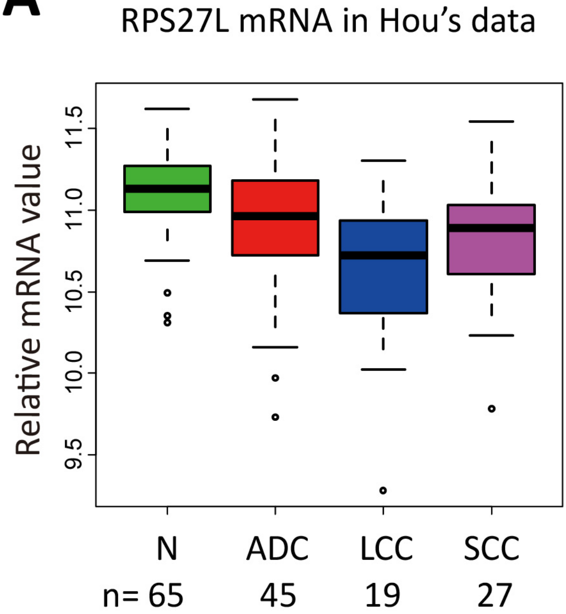

B

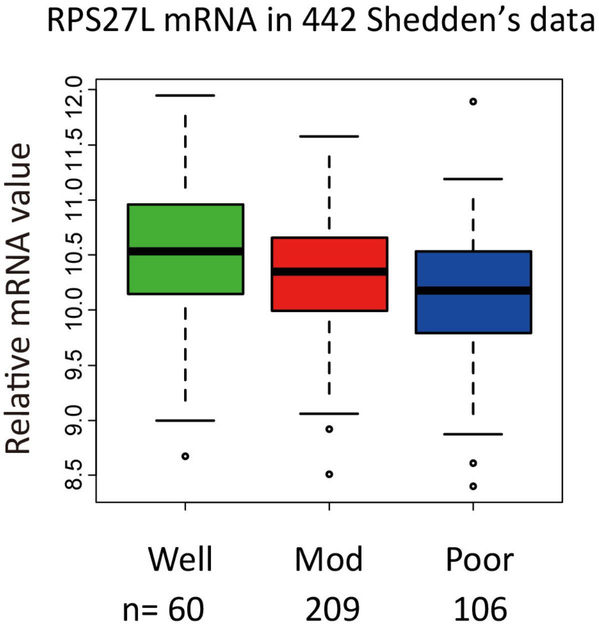

Supplement: Supplementary file 1 — Supplemental Materials [file 41419_2020_3082_MOESM1_ESM.pdf]
